# The Puppy in the Pit: Osteobiography of an Eighteenth-Century Dog at the Three Cranes Tavern, Massachusetts

**DOI:** 10.1007/s10761-021-00636-1

**Published:** 2021-11-11

**Authors:** Liz M. Quinlan

**Affiliations:** Archaeology, University of York, Environment Building, Wentworth Way, York, YO10 5NG UK

**Keywords:** Zooarchaeology, Osteobiography, Ritual, Dog burial

## Abstract

Boston’s “Big Dig” construction project resulted in the excavation of multiple archaeological sites dating from the seventeenth to nineteenth centuries, including the Great House/Three Cranes Tavern in Charlestown, Massachusetts (USA). An otherwise unremarkable pit below the tavern foundation contained bones originally identified as a cat skeleton, which has subsequently been reidentified as a dog. This paper discusses site context, osteological evidence for the dog’s reclassification, and the shifts in cultural meaning this may indicate. Employing an osteobiographical approach, it draws together points of connection between the modern skeletal assessment, a series of 1980s excavations, and the motivations of eighteenth-century tavern inhabitants.

## Introduction

**Fig. 1:**
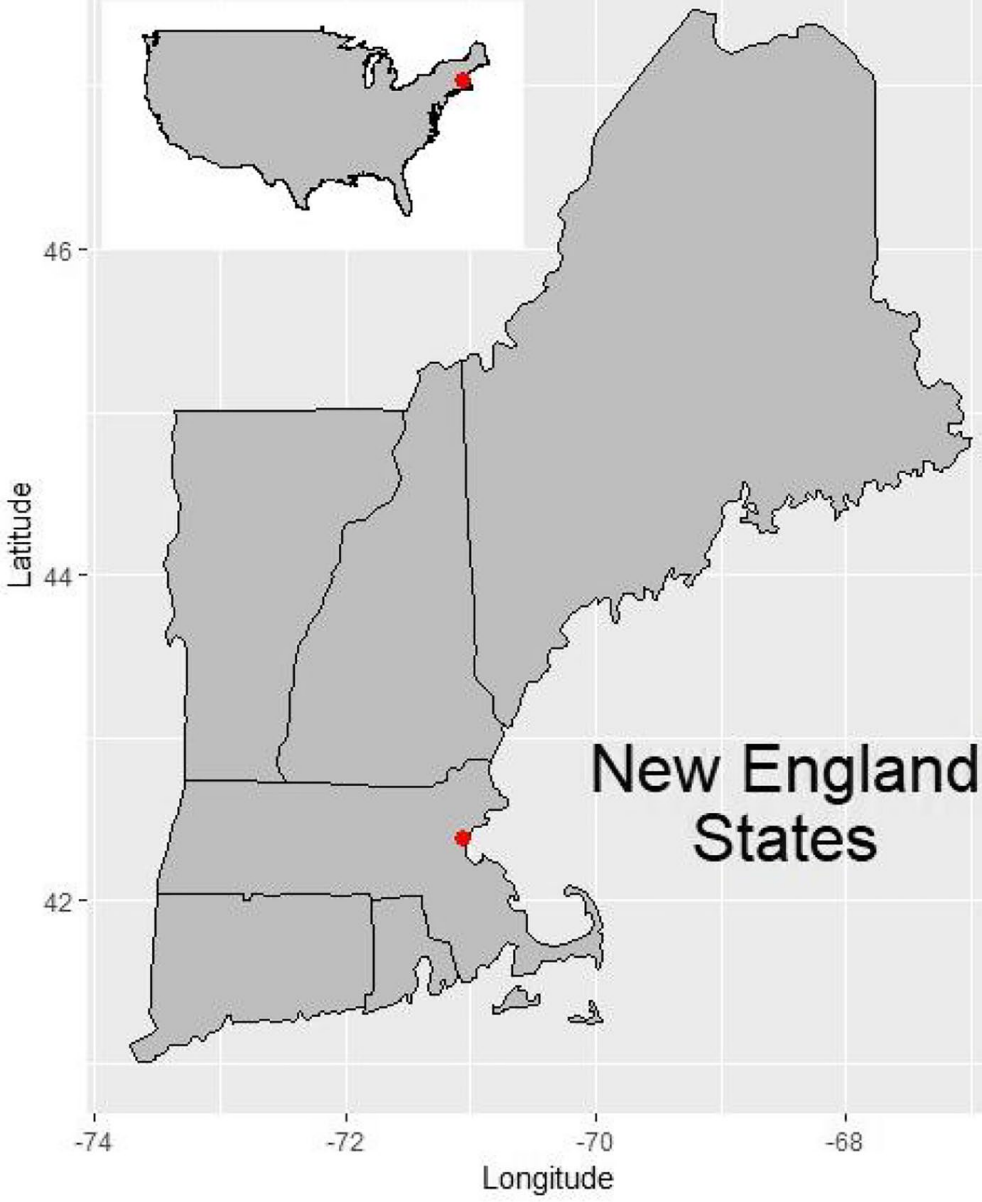
Map of the New England region of the Northeastern United States, with an inset map of the contiguous US. A red dot marks the location of Charlestown, Massachusetts (by author)

**Fig. 2:**
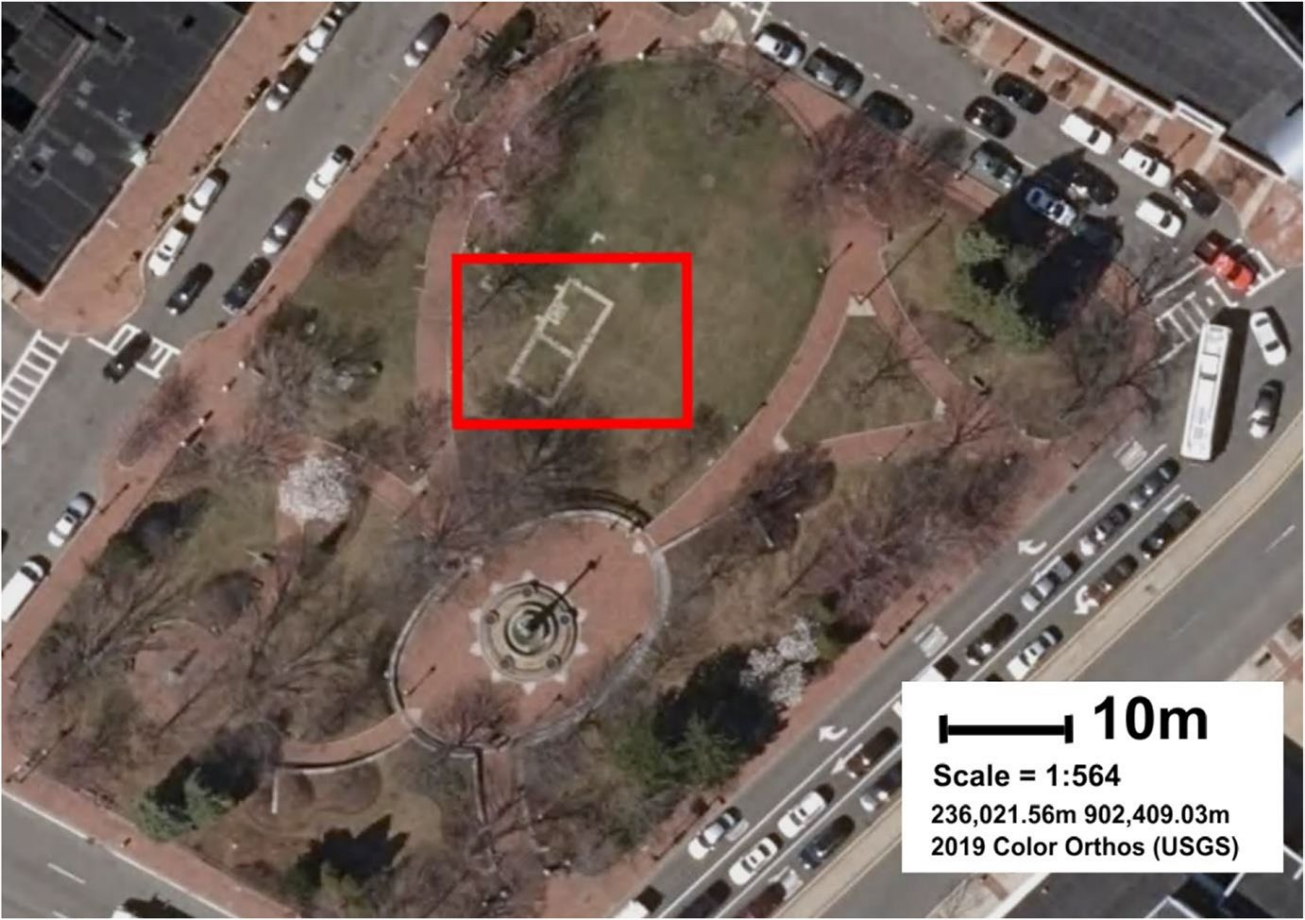
Satellite image of modern-day City Square, in Charlestown. City Square is a modern park located in the location of the City Square Archaeological District (CSAD). The red rectangle outlines the approximate location of the Three Crane’s Tavern and its associated buildings, marked with a paving stone outline in the park. Satellite image source: 2019 Color Orthos (USGS)

Excavations of the Central Artery North Reconstruction (CANR) Project in 1985 and 1987, led to the recovery of significant amounts of data regarding the seventeenth- and eighteenth-century settlement of Charlestown, MA (Fig. 1). Typical with urban archaeological efforts in the northeastern United States, the overall area was separated into individual sites, and varying degrees of post-excavation analysis has been conducted on each of them. One district within the CANR was the City Square Archaeological District (CSAD) (Fig. 2), which contained four individual sites within its boundaries: the Great House/Three Cranes Tavern (TCT), the Reverend Abbott House, the Ebenezer Breed House, and the Samuel Long House (Gallagher et al. 1994; Gallagher and Ritchie 1992). During the 1985 excavation, the entire CSAD site was treated as a single entity, however, separate assessments of assemblages recovered from each of the four sites have been produced since that time. The two primary sources of archaeological interpretation regarding the Great House/Three Cranes Tavern property are two 1992 and 1994 data synthesis reports produced by the excavators at the Public Archaeology Laboratory, or PAL INC (Gallagher et al. 1994; Gallagher and Ritchie 1992). The first, compiled by Joan Gallagher and Duncan Ritchie (1992) provides a synthesis of data and interpretations relating to the entire CANR project, while the second focuses specifically on the CSAD results (Gallagher et al. 1994).

The first reference to the species of an articulated skeleton recovered from the Three Cranes Tavern/Great House site is in the original CSAD excavation field notes, where the handwritten description of Level 11, Unit 42, Feature 193, states: “Higher up [in the level] there was still some debris and a nearly intact trailed slip on redware bowl and what seems to be a cat skeleton.” (Unknown Author 1985-87a).

From here, the identification of “cat” was repeated on plan records and other field notes, and appears to have been accepted as a definitive identification of *Felis catus*. However, no zooarchaeological specialist ever studied the “cat” skeleton from the Tavern site, and the identification was never questioned. After osteological analysis, detailed in this publication, it is apparent that this was a mistaken identification and the specimen instead belongs to the species *Canis familiaris*, or the domestic dog. Unfortunately, the misidentification became embedded in the literature and subsequent interpretations of the Three Cranes Tavern site assumed the animal was a cat. This report will explore site history, original identification and reassessment of the skeleton, and the cultural and archaeological impact of this new identification.

Over the last decade, several zooarchaeological investigations have taken an osteobiographical approach to investigating depositions of faunal material variously referred to as articulated skeletons, “special animal deposits” and “associated bone groups” (Grant 1984; Hill 1995; Morris 2011). Sometimes considered part of the “social zooarchaeology” movement (Ewonus 2011; Orton 2012; Overton and Hamilakis 2013; Russell 2011; Sykes 2014), osteobiography has its origins in bioarchaeology (Geller 2012; Hosek and Robb 2019; Robb 2002; Saul 1972; Saul and Saul 1989;). Osteobiography can be used to narrate life histories and sociocultural experiences through the bones of an individual, using taphonomy, pathology, and osteology to inform interpretations. The osteobiography of humans involves the “interaction of complex networks of circumstances” (Hosek and Robb 2019: 3), and when the method is applied to non-human animals these interactions are further complicated by the intricacy of human-animal relationships. Different animals occupy, and have occupied, varying positions of importance and influence across time and space- what one society considers a “pet,” another may see as unfit to be a human companion. Dogs straddle multiple levels of closeness to humans, filling “virtually every role in the whole spectrum of human-animal relationships” (Russell 2011:280). It stands to reason, then, that this uniquely situated species may occupy multiple roles throughout its life, regardless of whether it reaches an advanced age, or, as with the TCT dog, lives only a handful of months.

### Site History

This skeleton was uncovered during the 1985–87 CSAD excavations of the Great House/Three Cranes Tavern in Charlestown, Massachusetts. The central location of the Three Cranes Tavern/Great House in Charlestown made it an important center of economic and political activity in the newly chartered Massachusetts Bay Colony (Gallagher et al. 1994; Gallagher and Ritchie 1992; Lewis 2001). The “Great House” was constructed in 1629 and originally it was intended to be both a residence for the first elected governor of the colony, John Winthrop, and house the chambers for the general Court of the Massachusetts Bay Colony (Gallagher et al. 1994; Lewis 2001). However, after only a few months of use the property was abandoned by the governor and eventually sold by the Bay Company to the General court in 1632. The reason for its sale and abandonment is not entirely clear, but it is possible that a series of deaths among settlers were attributed to the increasingly brackish waters of Charlestown, leading the colonial government to relocate across the Charles River to present-day Boston (Frothingham 1845:31; Gallagher et al. 1994; Young 1846). By 1635, it had been purchased by Robert Long, opened as a lodging and public house, and officially named the Three Cranes Tavern (Gallagher et al. 1994; Gallagher and Ritchie 1992). Members of the Long family owned the property until it was transferred to Nathaniel Brown in 1746 (Gallagher et al. 1994; Gallagher and Ritchie, 1992). During his ownership, substantial repairs were made to the then 117-year-old building, including a new stone foundation built on the southern side (Gallagher et al. 1994). Around the time of these renovations, the body of a small domesticated mammal was placed in an intentionally dug pit underlying the new cobble foundation, designated as Feature 193 in the 1985–87 CSAD excavations.

### Tavern Construction and Burial Context

To understand the context in which the TCT dog was buried, the tavern culture and its various building periods need to be explored further. The 1994 Gallagher et al. report synthesized a timeline of the archaeological site from its original 1629 Great House structure to the TCT that was destroyed by British troops during the June 17, 1775 Siege of Boston. Ownership of the property and some of the structural alterations over time can be traced via probate and other public records, as well as through archaeological evidence. The 1994 site report concludes that after the property was passed to Samuel Long from his mother Mary in 1711, a period of extensive structural changes began (Gallagher et al. 1994). Samuel Long’s major contribution was a new house addition, called the Long House, added to the northeast corner of the tavern building. At death the property passed to his widow Sarah Long Shore, undergoing a period of occupation by tenants. After Sarah’s death in 1743, the property was then sold to Chambers Russell and, eventually, transferred to Nathaniel Brown in 1746. Brown owned the Tavern for 30 years and it appears that it was during his ownership that the extensive repairs which involved the TCT dog deposition occurred. Gallagher et al. (1994:168, 190) list the repairs made between 1746–75 as follows:

- Small cellared addition on northeast side of house;
- New brick floor laid around hearth;
- Addition and stone foundation on south side of tavern;
- Extension onto northeast side of tavern;
- Cobbled areas put in south side of tavern’s yard for drainage; and
- Four of the five privies on the lot built.

In 2016, archaeologist Craig Chartier conducted an architectural reassessment of the Great House/Three Cranes Tavern property. Chartier (2016) referenced the 1992 and 1994 site report conclusions about the position of the buildings and sequence of construction, and focused mainly on seventeenth-century vernacular architecture and his disagreements with original interpretations. Minutiae about seventeenth-century vernacular architecture styles and interpretation aside, Chartier does offer a bit of clarification on the orientation of the TCT, providing evidence for a southeast tavern entrance and interprets the dog skeleton as being placed directly under a new threshold added during the eighteenth-century renovations. It is suggested by both Chartier and the original Gallagher et al. report that the “cat” in Feature 193 was most likely deposited in the first half of the eighteenth century either under an existing ephemeral porch or directly in front of the front door, and the Nathaniel Brown-era tavern additions were built over it (Chartier 2016; Gallagher et al. 1994).

### Feature 193 Contents

**Fig. 3:**
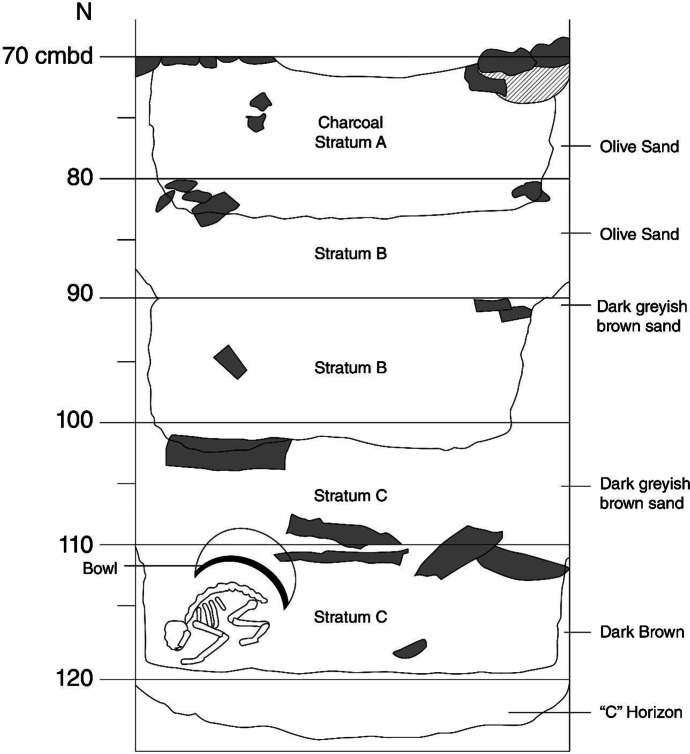
Profile drawing of Feature 193, showing stratigraphic change throughout the feature and indicating the approximate location of the dog skeleton and redware bowl. Artifact and architectural features are derived from feature plans and excavation notes, and are not necessarily to scale. Unit was excavated in 10-cm arbitrary levels, and Feature 193 was overlain by Feature 89, the stone foundation addition to the tavern seen in the northwest corner of the image). Image source: author and Micaela Brody

Feature 193 (Fig. 3) is a self-contained pit feature identified by excavators in the northwest quadrant of Unit 42, a 2 m x 2 m square excavation unit. Commercial excavation in the northeastern US at the time resulted in descriptions of 10-cm arbitrary levels, natural and anthropogenic strata, and natural soil horizons. Soil horizons are based on USDA National Soil Survey Center descriptions of three master horizons; the surface (A horizon), subsoil (B horizon), and substratum (C horizon) (Schoeneberger et al. 2012). Unfortunately, the CSAD excavation also used letters A-C to refer to changes in soil characteristics, resulting in Feature 193 having Strata A, B, and C, as well as a C Horizon. Excavation of Unit 42 began with removal of topsoil and organic material, at which point starting measurements for Level 1 were taken at each corner of the 2 m x 2 m unit. The rest of Level 1 was then brought down to the depth of its lowest starting corner depth, to create a level surface for the beginning of Level 2. Levels 2–12 of Unit 42 were then excavated in 10-cm segments.

There is some disagreement between the field notes and plan records regarding the exact dimensions of Feature 193. The excavator’s field notes describe a circular pit with a total depth of 60 cm and horizontal dimensions of 80 cm x 80 cm, from the bottom of Layer 6 to the top of Layer 12 (Unknown Author 1985-87a). The plan records, which were used to reconstruct a profile drawing of the feature (Fig. 3), describe the pit as being just under 50 cm deep (from roughly 70-118 cm bd), with the same 80 cm x 80 cm horizontal dimensions (Unknown Author 1985-87b). Both sources describe the feature as having fairly straight sides, a flat bottom, and several fill sequences, ranging from charcoal to various sandy and clayey sediments and no evidence of wooden barrel remains were noted in either the original field notes or final site reports. The excavator also notes that Feature 193 “passes underneath F. 89, a stone wall,” which was later also described as part of the Great House addition foundation/floor joist support, added somewhere between 1746–75 ( Gallagher et al. 1994; Gallagher and Ritchie 1992; Unknown Author 1985-87a: 3). Reading Feature 193 from the top down seems to show two later cuts (Strata A and B) into the original pit feature (Stratum C), perhaps used as builder’s trenches or rubbish dumps during the tavern addition’s construction period. In the handwritten field notes the excavator states that within Level 11, where the skeleton was found, there is a near complete absence of building materials, which had been present in previous layers.

The skeleton itself was located at a depth of between 110 and 120 cm and may have been associated with a small, mixed faunal and ceramic assemblage. There were 27 animal bone fragments and seven ceramic artifacts recovered from between the top of Level 10 and bottom of Level 11. Post-excavation notes list 16 mammal, four bird, one fish, and six indeterminate bone fragments, as well as three redware sherds, one kaolin pipe bowls, and three kaolin pipe stems. The animal remains were not identified beyond class, the ceramic sherds were only superficially described, and both are currently inaccessible for further study at the time of publication. The pipe stem sizes were recorded using the bore diameter and all three had Size 5 (5/64 in or 1.98 mm) bore sizes. This size classification is based on a pipe stem dating system described by Deetz (1999), which used drill bits ranging in size from 9/64 in (3.57 mm) to 4/64 in (1.59 mm), in order to standardize pipe stem bore descriptions. This system has been shown to accurately date pipe stems, with a 5/64 in (1.98 mm) bore corresponding to a manufacture date range of 1720-50 CE (Deetz 1999).

Based on clarifications in the field notes and final site reports, it appears that the articulated skeleton should be considered a separate deposit from the other scant faunal remains found in Feature 193. The field notes (Unknown Author 1985-87a, 1985-87b) indicate that the 27 fragments of bone material may be associated with one of the aforementioned secondary deposits cuts made in Level 10, or even 9, while the articulated dog skeleton was found close to the bottom of Level 11, and closely associated with a redware bowl. This slip-decorated redware vessel (Fig. 8) is of particular importance, as it is referred to as being associated with the skeleton by all researchers (Bagley 2016; Chartier 2016; Gallagher et al. 1994; Gallagher and Ritchie 1992; Lewis 2001). It is dated to between 1725 and 1740, and is an example of locally produced Charlestown slip-decorated redware, potentially made at any one of the neighboring Charlestown potteries, including the Parker Harris pottery located adjacent to the CSAD (Gallagher et al. 1994; Gallagher and Ritchie 1992). This 1725–50 ceramic date, the 1720–50 pipe stem dates, and the construction sequence resulting in a new stone foundation dated to between 1746–75 (Feature 89) stratigraphically above Feature 193, all point to a deposition date after 1725, but before 1775, and perhaps even before 1746. The TCT dog was most likely deposited in its pit either just before or during the period when Nathaniel Brown made his numerous additions to the Great House property.

## Osteological Analysis

**Fig. 4:**
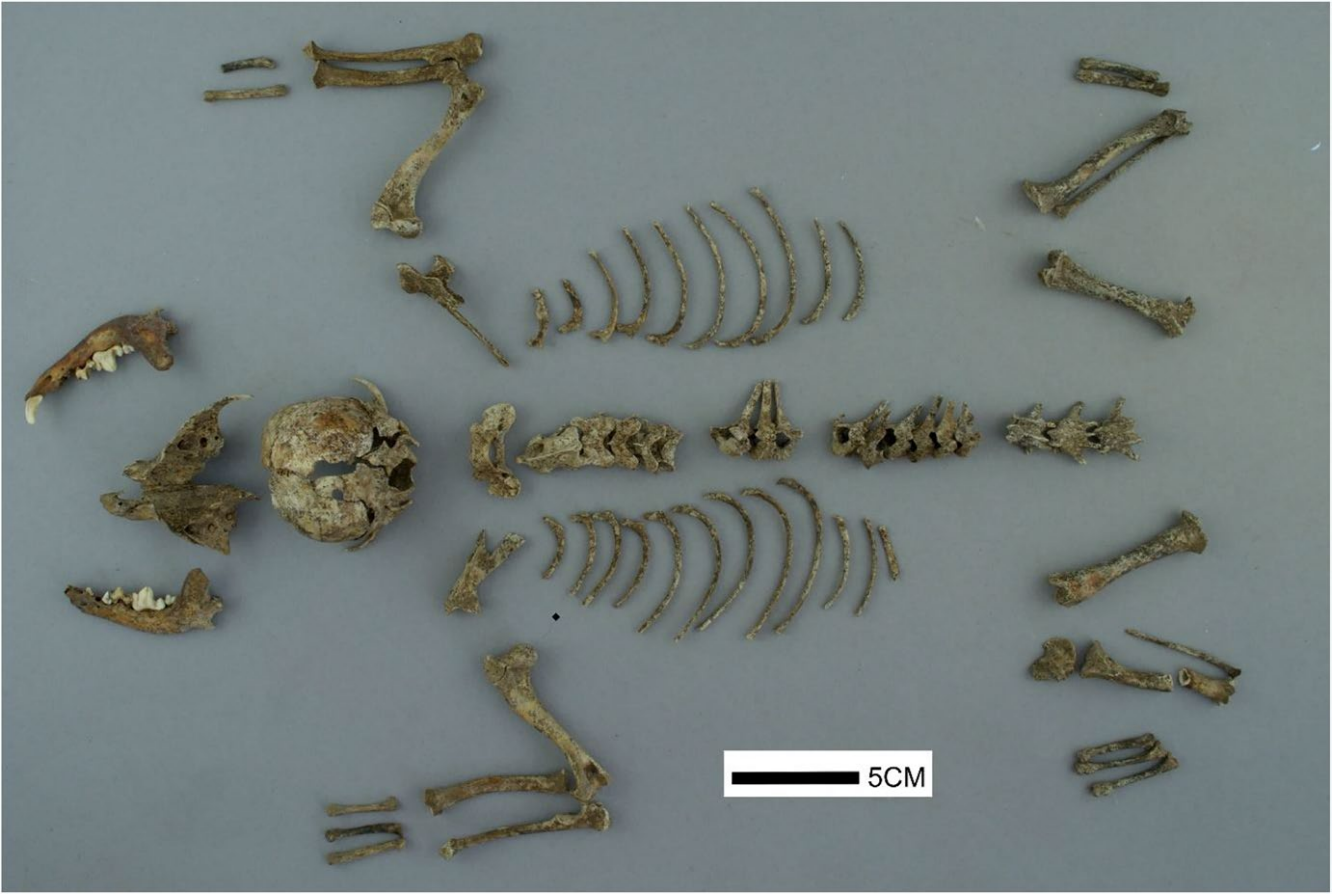
Top view of the Three Cranes Tavern dog, showing all recovered elements (by author)

The assessment of this specimen was conducted using two main methods of analysis; faunal comparison and osteometrics. The Three Cranes Tavern (TCT) specimen (Fig. 4) was directly compared to three dogs, two cats, two fox, and one raccoon specimen obtained from the type collection at the University of Massachusetts’ Fiske Center for Archaeological research. All reference skeletons were adults, with no significant pathologies present. One dog was from a pug-type brachycephalic breed, and the other two were medium-sized animals of an unknown breed. In addition to this direct comparison, multiple texts, articles, and online resources were used for both initial identification, size and age estimation (Andreis et al. 2018; Coulson and Lewis 2002; Done et al 2009; Evans and De Lahunta 2012; Geiger et al. 2016; Hillson 1990, 1992; Lewis 2019; Modina et al. 2019; Onar and Belli 2005; Onar et al. 2013). The main focus of the reassessment was a comparative analysis between dog and cat anatomy, focused on dentition, presence/absence of a supracondyloid foramen, cranial shape, appendicular skeleton, and body size estimates. Osteometric data was obtained from all present portions of the appendicular skeleton, and present portions of the cranium and mandible, and recorded following measurements outlined in Von den Dreisch (1976). A cursory review of skeletal pathology was conducted, and preliminary observations are discussed later in this section.

### Comparative Cranial and Dental Analysis

The importance of dental analysis in this case is twofold; not only does it offer evidence to support a conclusive species identification, it also offers information on the age of the individual. Dentition is the steepest contradiction to the previous identification, as this individual’s present teeth do not conform to the typical feline dental formula (Hillson 1992). Even while missing several mandibular and maxillary premolars, the lower left canine, and all incisors, this specimen conforms to the canid formula. Table 1 describes all present teeth; 17 out of a possible 42. There is some post-mortem breakage, possible pre-mortem abrasion and/or breakage, and general discoloration visible across the present maxillary and mandibular teeth. There is also a significant amount of tooth crowding seen in the maxillary and mandibular molars and premolars of this specimen.

**Table.1:** Condition and Location of all Present Teeth

Tooth	Triadan Number	Condition
Right maxillary canine	104	Some discoloration; cracking; complete
Left maxillary canine	204	Some discoloration; cracking; complete
Right maxillary P4	108	Complete
Left maxillary P4	208	Soil staining/yellow discoloration; complete
Right maxillary M1	109	Some discoloration, complete
Left maxillary M1	209	Complete
Left maxillary M2	210	Some discoloration; complete
Left mandibular canine	304	Some discoloration; lateral splitting, possible enamel hypoplasia
Right mandibular P2	406	Some discoloration; possible abrasion, complete
Right mandibular P3	407	Post-mortem breakage
Right mandibular P4	408	Soil staining/yellow discoloration; post-mortem breakage; possible enamel hypoplasia
Left mandibular P4	308	Soil staining/yellow discoloration; possible abrasion and/or breakage; possible enamel hypoplasia
Right mandibular M1	409	Lateral cracking; complete
Left mandibular M1	309	Lateral cracking; complete
Right mandibular M2	410	Complete
Left mandibular M2	310	Complete

**Fig. 5:**
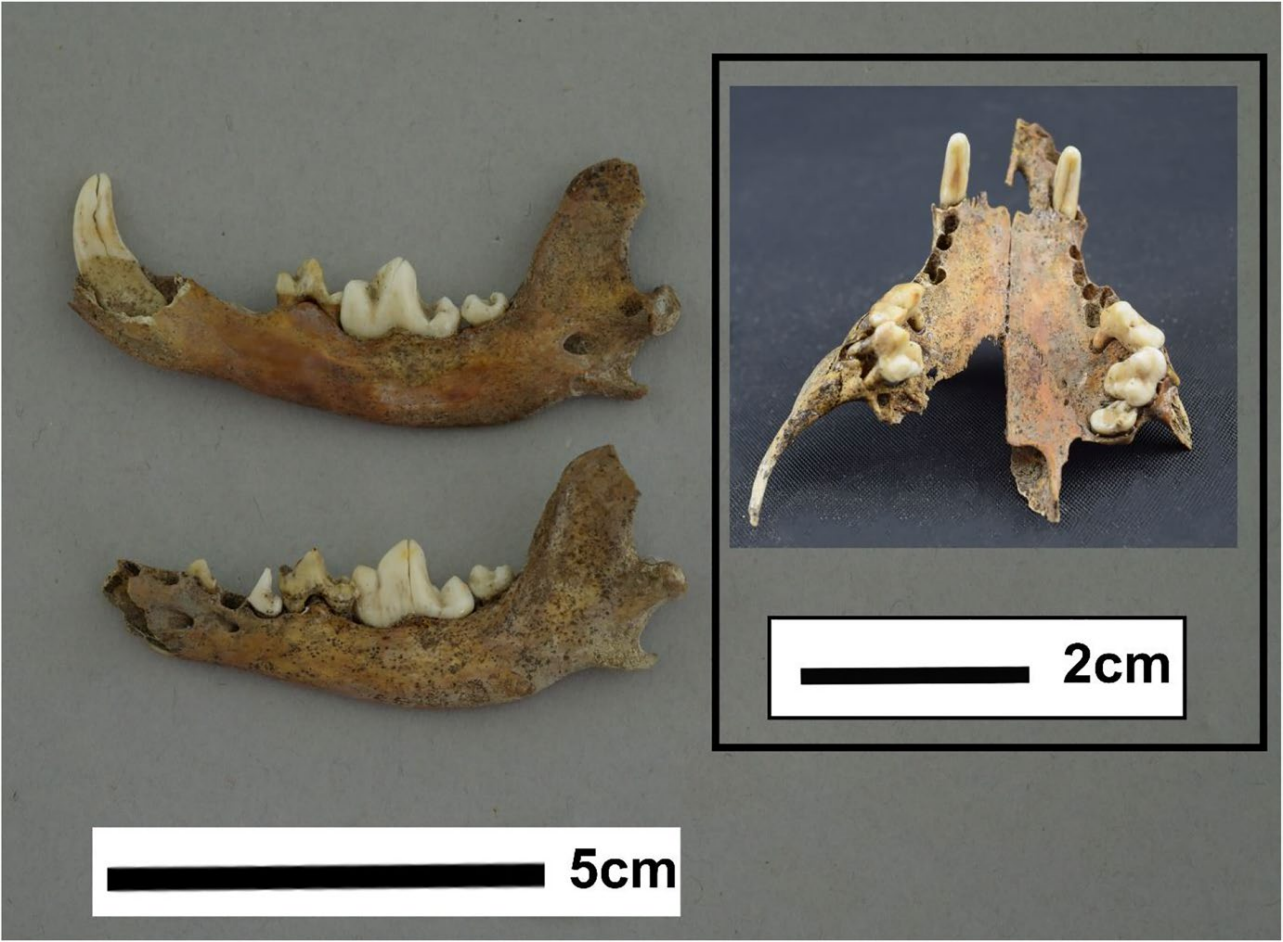
Image of the TCT dog mandibles and maxillary arcade, showing all existing teeth (by author)

The existing teeth and remaining alveoli present in both the mandibular and maxillary dental arcade are congruent with the 3/3 I, 1/1 C, 4/4 P, 2/3 M permanent dental formula of the domestic dog (Hillson 1992) (Fig. 5). In dogs, incisors and canines generally erupt between 3–4 months, premolars between 4–6 months, and molars between 5–7 months on average (Fulton et al. 2014). The TCT dog does not have a present left or right mandibular M3–typically the last of the molars to fully erupt – nor are there alveoli for the teeth, which may be indicative of congenital anodontia. A recent survey of anodontia in domestic dogs and cats showed that 7.8% (n = 8) of the unerupted teeth reviewed were mandibular M3s, and, of those mandibular M3s, the vast majority were from small breed dogs (Bellei et al. 2019). So, while rare, a missing M3 is certainly not unheard of and may actually be more likely due to the size of the TCT dog. The possibility that this missing M3 is congenital limits dental aging to an unfortunately vague assessment of older than five months.

While dental analysis has conclusively proven that the individual was not a cat, it is important to rule out coyotes, wolves, red foxes and raccoons—other mammals endogenous to Massachusetts. Direct comparisons were made to *Vulpes vulpes* and *Procyon* *lotor* specimens, and then eliminated based on incompatible cranial and dental morphology. There have been numerous attempts to standardize identification of canids based on cranial and dental differences over the years (e.g., Ameen et al. 2017; Andersone and Ozolins 2000; Benecke 1987; Bockelmann 1920; Clutton-Brock 1963; Germonpré et al. 2015; Morey 1994; Olsen and Olsen 1977; Studer 1901). However, recent reviews and morphometric studies of these collected methods concluded that many measurements used for differentiation show a great deal of variation within modern and archaeological dog, wolf, coyote, and fox specimens (Janssens et al. 2019; Welker et al. 2020). A mulitvariate approach is best for distinguishing dogs from other canids, and in this case the ratio of M1 length to mandibular bowing in the TCT dog specimen was compared to the measurements of the same presented in Welker et al. (2020). While the TCT dog specimen’s left M1 length of 13.66 mm and mandibular bowing angle of 17.17° falls outside the significant cluster (95% CI) of domestic dog measurements presented by Welker et al. (2020), it does closely correspond to several samples within their dataset which belong to both Chihuahua and toy sized mixed breed dogs (Welker et al. 2020).

**Fig. 6:**
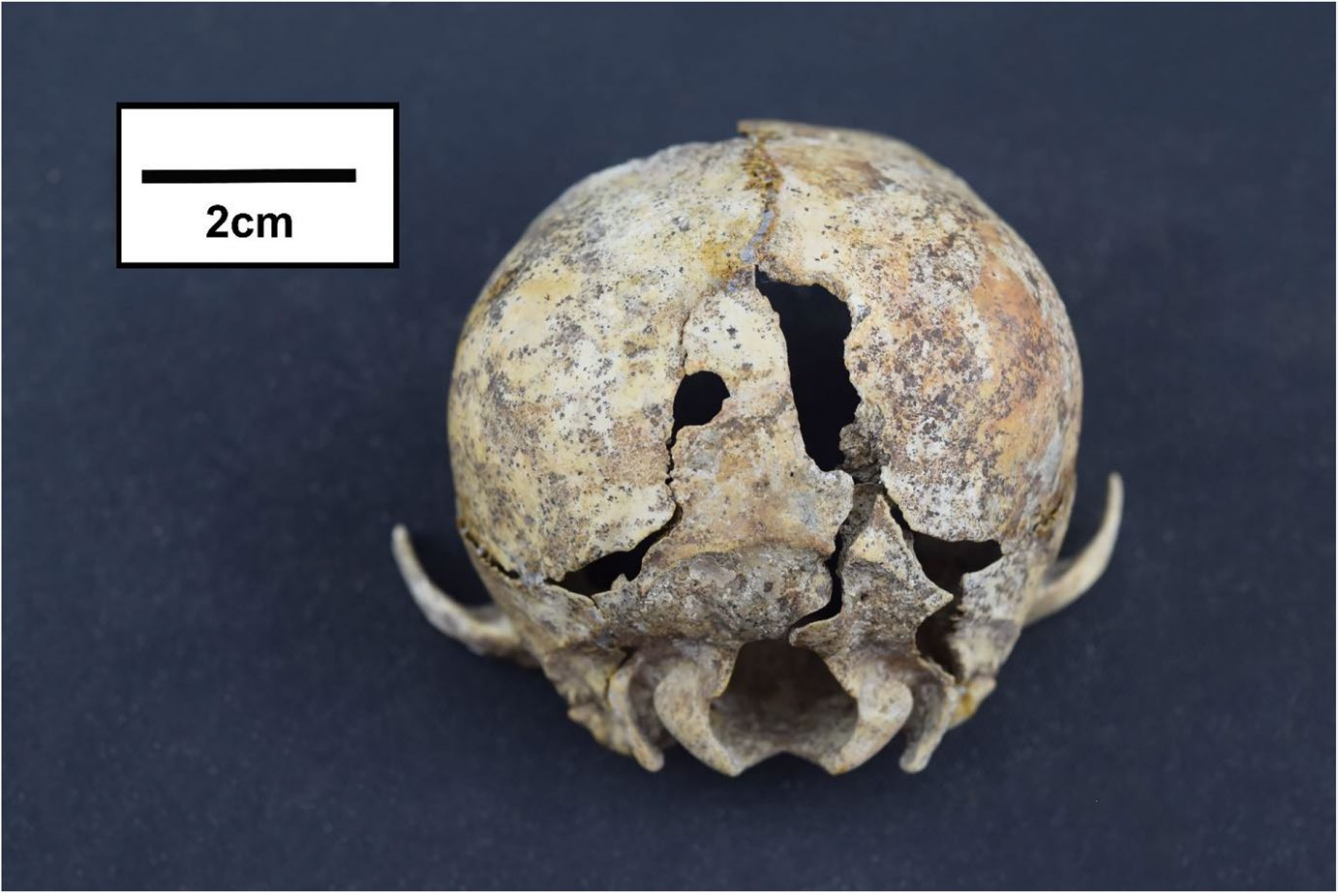
Posterior view of the skull, showing the constrained tympanic bullae, dorsal notching of the foramen magnum, and evidence of blunt force perimortem trauma (by author)

Still in the middle of its skeletal development, this specimen displays some features that serve to confuse initial observation, including its rounded cranium, comparatively prognathic rostrum, lack of a protruding external sagittal crest, and mostly absent orbital area. It is initially difficult to determine if the skull may have the typically large and rostrally positioned orbits of a cat, or if its fragmentary snout might have initially shown the straighter, narrower nasals of a dog (Done et al. 2009). While differences between adult dog and cat skeletons are readily discernible, developing dogs and cats go through rapid morphological changes that can explain some of these contradicting features (Done et al. 2009; Evans and De Lahunta 2012; Newton and Nunamaker 1985). Additionally, there are aspects of this individual’s cranium that clearly identify it as a dog skull. Figure 6 shows a posterior view of the TCT skull, with a distinctive dorsal notch in the foramen magnum, which can be typical of small mesaticephalic or brachycephalic breeds of dogs (Evans and De Lahunta 2012; Kupczynska et al. 2017; Onar et al. 2013), and its constrained tympanic bullae, which are smaller and less bulbous than those in cats (Done et al. 2009; Evans and De Lahunta 2012). When directly compared to both a dog and a cat skull, the TCT skull clearly has more in common with the canine specimen; it lacks the domed nasal bones and constricted rostrum of the cat, and shows the more proximal infraorbital foramina of a dog (Done et al. 2009; Evans and De Lahunta 2012; Hillson 1992).

### Appendicular Skeleton

While basic anatomy of the humerus in domestic dogs and cats is fairly similar, there are two major anatomical differences between the humerus of the two species: the presence of a supracondylar (sometimes rendered as “supracondyloid”) foramen in cats and the presence of a fully perforated supratrochlear foramen in dogs (Evans and De Lahunta 2012; Harasen, 2009; Newton and Nunamaker1985; Stefanowski and Zablocki 1969). In cats, the supracondylar foramen is responsible for guiding the median and brachial nerves along the condylar surface of the humerus and, according to a study conducted by Polish anatomists Tadeusz Stefanowski and Jacek Zablocki (1969), was present in at least one humerus of all domestic cats studied. While there can be thinning of bone in the olecranon fossa of cat humeri, there is no true supratrochlear foramen (Newton and Nunamaker 1985). It is clear from direct comparison that not only is there a fully formed supratrochlear foramen, there is also no evidence of a supracondylar foramen on the TCT specimen. Neither the left nor right humerus of the TCT specimen show evidence of a supracondylar foramen and it can be concluded that the humeri of the individual do not belong to a cat but a dog.

The appendicular skeleton of this specimen was essential in determining an approximate age at death. Using osteological and radiological data on observed fusion times in canine appendicular skeleton a more accurate estimate of age at death for the TCT dog was obtained (Table 2). Based on observed fusion in the TCT dog it seems likely that it was between 6.5–7.5 months old at time of death, with the proximal humerus fusion state acting as a limiting factor in age determination.

**Table.2:** Variation in Canine Appendicular Fusion Rates and Observed Fusion Status of Secondary Ossification Centers on TCT Dog

Element	Secondary Ossification Center	Earliest time of fusion (Sumner-Smith 1966)	Latest time of fusion	Fused in TCT Dog	TCT Age Estimate (Coulson and Lewis 2002)
Scapula	Scapular tuberosity	12 weeks	5 months	Yes	> 17 weeks
Humerus					
	Proximal epiphysis	10 months	10 months	No	< 30 weeks
	Distal epiphysis	5 months	8 months	Yes	25–34 weeks
Ulna					
	Proximal epiphysis	5 months	8 months	Yes	25–34 weeks
	Distal epiphysis	6 months	8 months	Yes	25–34 weeks
Radius					
	Proximal epiphysis	5 months	8 months	No	< 34 weeks
	Distal epiphysis	6 months	9 months	No	25–34 weeks
Metacarpal					
	Proximal epiphysis	5 months	7 months	Yes	> 17 weeks
	Distal epiphysis	5 months	7 months	Yes	> 17 weeks
Femur					
	Proximal epiphysis	6 months	9 months	No	
	Distal epiphysis	6 months	8 months	No	
Tibia					
	Proximal epiphysis	6 months	11 months	No	
	Distal epiphysis	5 months	8 months	No	
Fibula					
	Proximal epiphysis	6 months	10 months	No	
	Distal epiphysis	5 months	8 months	No	
Metatarsal					
	Proximal epiphysis	5 months	7 months	Yes	
	Distal epiphysis	5 months	7 months	Yes	

Osteometric data derived from the few fused limbs of this skeleton allows for a life estimation of withers height, using Harcourt’s (1974) regression formula. Table 3 displays the data estimations of withers height, with an average calculated withers height of 19.35 cm at between 6.5–7.5 months of age.

**Table.3:** Withers height estimation for the Three Cranes Tavern dog skeleton

Element Greatest	Length (GL) ***(mm)***	Regression Formula Estimated Height at	Withers (cm)
Left Humerus	65.01	((3.43x (TL) – 26.54)/10	19.64
Right Humerus	65.46	((3.43x (TL) – 26.54)/10	19.79
Left Ulna	65.36	((2.78xTL) + 6.21)/10	18.79
Right Ulna	66.73	((2.78xTL) + 6.21)/10	19.17
***Average***			19.35

### Trauma and Skeletal Pathology

One very noticeable feature of this individual’s cranium is the perimortem trauma on the occipital and parietal bones (see Fig. 6). While the skull and several other bones of this skeleton show postmortem changes consistent with taphonomic diagenesis and excavation damage (Hedges 2002), these locations on the occipital and parietals are the only clear instance of perimortem trauma. The smaller area of damage on the left parietal-occipital border measures 5.1 mm at the widest and 5.3 mm at the longest. The larger area of damage on the right, located more dorsally, measures 20 mm at the longest and 6.9 mm at the widest. Both areas of damage show no evidence of antemortem healing, and show the characteristic obtuse angles and smooth texture of blunt perimortem injuries (Calce and Rogers 2007; Kranioti 2015; Moraitis et al. 2009). Without any other skeletal perimortem damage, it appears that these instances of blunt force trauma relate to the cause of death for the TCT dog.

Unlike human forensic pathology, there have been very few investigations into the differences between accidental and non-accidental traumatic skeletal injuries in dogs (Gerdin and McDonough 2013; Ressel et al. 2016). However, the size and shape of the injuries observed on this skull may indicate intrusion with a sharp implement like an axe or knife, a projectile like lead shot, or a fall onto a protruding sharp edge or surface. Fall trauma is difficult to discuss in this circumstance, as little work has been done on the characteristics of fall-derived trauma in animals compared to that done on blunt force trauma and gunshot wounds (Gerdin and McDonough 2013). A 2016 veterinary pathology study of sharp injuries in domesticated animals produced an example of a postmortem chop mark made with an axe on a dog’s skull that bears a striking resemblance to the damage seen on this right parietal-occipital border of the TCT dog’s skull (de Siqueira et al. 2016). Similarly, the left parietal-occipital border damage resembles the trauma inflicted by gunshot wounds (Moraitis et al., 2009; Yasar 1998). In this case the projectile would have to be quite small, but there is ample evidence for small calibre shot being used in the eighteenth-century, especially for bird hunting (Breen 2013; Smith 2014). While these examples clarify potential causes of death, the motivation remains unclear.

Some features of the TCT skeleton indicate developmental issues that may relate to severe malnutrition, disease tropisms, or congenital disorders. Physical disability or illness is one compelling motivation for killing a young dog that could have become a valuable hunter, companion, or guard dog. Though dorsal notching in the occipital bone is a known feature in small dogs (Evans and De Lahunta 2012; Kupczynska et al. 2017; Onar et al. 2013), it can also be indicative of a Chiari-like Malformation (CM) (Knowler et al. 2018). CM in dogs can present with mild to severe neurological effects, ranging from head-shaking and neuropathic pain, to proprioceptive and vestibular dysfunction (Kupczynska et al. 2017; Rusbridge et al. 2019). If some of the more severe clinical symptoms were present in this dog, it may have been killed due to a perceived inability to function normally.

**Fig. 7:**
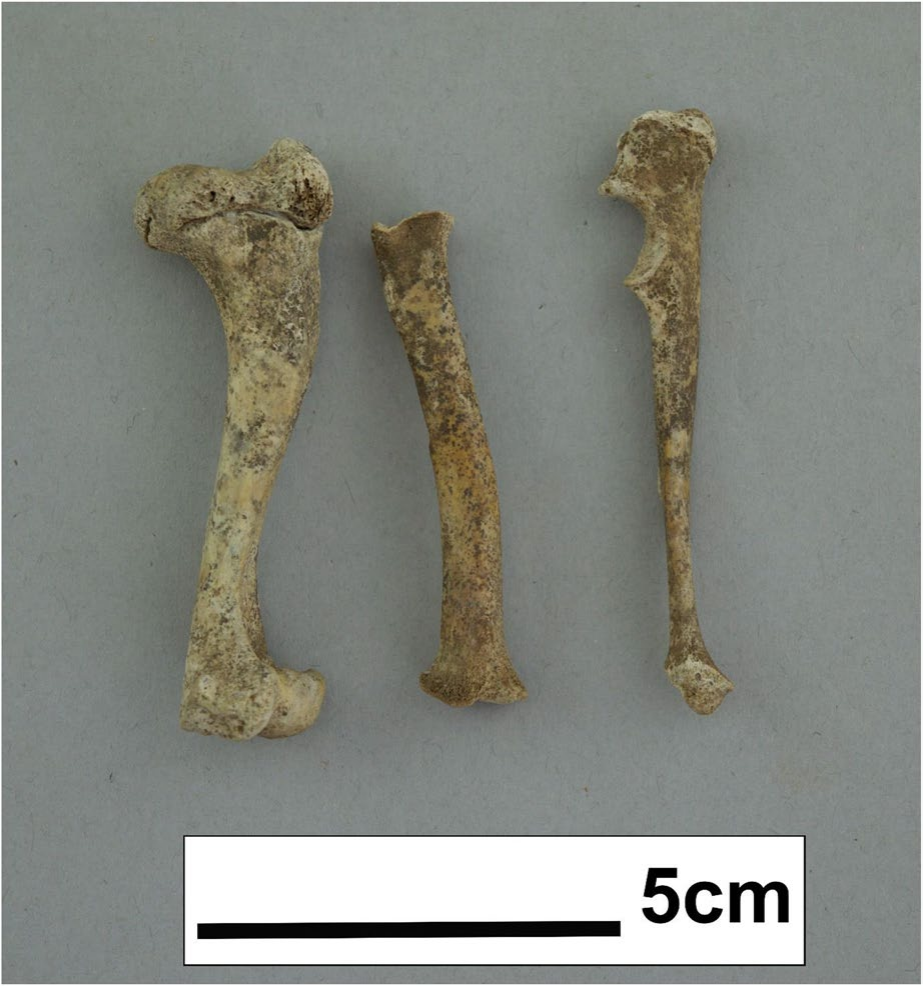
Bones of the right forelimb (humerus, radius and ulna), showing bowing typical of several possible congenital and nutritional disorders (by author)

As mentioned in the earlier dental section, several of the present teeth showed possible evidence of disease and wear. The virus responsible for canine distemper is known to cause developmental dental abnormalities, like enamel hypoplasia, impacted teeth/crowding, and porous alveolar walls – all seen within the TCT dog. However, canine distemper, and another common puppyhood illness which can cause dental abnormalities, parvovirus, was not present in eighteenth-century Massachusetts (Carmichael 2005; Uhl et al. 2019). Similarly, dog-maintained rabies was virtually absent from the Western Hemisphere until the late eighteenth century, and even then most cases were restricted to South America (Tarantola 2017; Velasco-Villa et al. 2017). However, the first rabies epidemic in North America was reported in Boston in 1768, within the 1725–75 period when the dog was likely buried, making rabies a remote, but possible, reason for the dog’s death (Kerr and Stimson 1909). If this was the case, the Three Cranes Tavern dog may represent an early case of dog-maintained rabies in Massachusetts. In addition to viral or bacterial disease, malnutrition-related disorders, such as rickets, are quite common in young dogs fed unsuitable diets, and often results in abnormal bone growth in dogs affected if the diet is not corrected (Grunberg 2018). The TCT dog does appear to display some bowing and warping of various long bones (see Fig. 7), which may, along with some of the dental pathologies observed, be indicative of insufficient diet, chondrodystrophy, or a combination of both.

This current discussion and postmortem diagnosis of specific diseases or disorders that may have affected the TCT dog is purely speculative, and would benefit from full palaeopathological and isotopic analysis. This extended analysis would help narrow down some of the proposed mechanisms for skeletal abnormality observed in the TCT dog, especially regarding nutrition and juvenile diseases. The ongoing COVID-19 pandemic has, unfortunately, limited these possible analytical avenues for the moment, but illness remains a potential explanation for the killing of the TCT dog. This explanation may be independent of, or combined with, potential spiritual or cultural meaning assigned to the deposition of the dog within the Feature 193 pit at the Tavern.

### Breed Determination

Selective breeding has led to incredible physical variation within dogs, resulting in hundreds of recognized breeds, each with distinctive physical traits. An additional factor complicating the initial identification of the TCT dog relates to the fact that most faunal collections and illustrations that archaeologists utilize present dogs such as the Golden Retriever or German Shepherd Dog as the domestic dog “type” – which can lead to variations in skull type being overlooked. Due to its developmental stage, fragmented nature, and damage to the cranium, a complete suite of cephalic index measurements was impossible in this case. Cephalic measurements classify skulls as dolichocephalic (long-headed), mesaticephalic (middle-headed), or brachycephalic (short-headed) (Evans and De Lahunta, 2012; Stockard et al. 1941). Though the TCT dog cannot be confidently assigned to a breed group from cephalic measurements, there are other indicators in general skull morphology, body size, the possible chondrodystrophy, historical references, and geographic location. Although historical breeds differed significantly from the breed standards of today, it is possible to use the body size standards put forth by these kennel club organizations to determine some various breed types that this individual may have belonged to. Given small dogs reach maturity earlier than larger breeds (Lewis 2019), it can be inferred that this specimen was about one–two months of growth away from its adult size. This allows for comparison to some generalized dog types of the eighteenth century.

Terriers as a group were reported as early as the sixteenth century. John Caius (2008 [1570]:5) identifies the *terrar* dog in his 1570 work “De Canibus Britannicis,” describing them as dogs which “creepe into the grounde, and by that meanes make afrayde, nyppe, and byte” small ground dwelling game.

From this general group came specialized breeds including small, agile terriers specially bred to deal with vermin. Rat terriers have existed in England in some form since the sixteenth century, with the most famous example being “Hatch,” the ship’s dog recovered from the wreck of the *Mary Rose* (Zouganelis et al. 2014). Hatch has been extensively studied, is postulated to be a terrier-type dog and a potential genetic precursor to the modern Jack Russell Terrier (Zouganelis et al. 2014). North American terrier-type dogs developed from possible admixture between Indigenous and European dog breeds, including those referred to as “feists,” “fyce,” or “foist” dogs (Donald and Stotik 1992). This “feist” dog was initially a generic term for a small dog, often used derogatorily for feral dogs of unknown history – as when George Washington (1770) referred to one of his prize female hounds, Countess, escaping and being bred twice by a “small foist looking yellow cur.” Now recognized as a distinct breed, the American Mountain Feist is described as a “treeing” dog, and the eighteenth-century progenitors of this breed would have been used for similar hunting strategies (Donald and Stotik 1992). Parallel to the development of these working breed groups was the creation of “toy” dogs intentionally bred for smaller stature and generally kept as companions or pets. Toy breeds like the Pomeranian, King Charles Cavalier Spaniel, Maltese, Phaléne, and the now-extinct Toy Bulldog were all well established by the mid-late eighteenth century (Blunt-Lytton 1911; Caius 2008 [1750]; Drury 1903; Shaw 1881). Many of these toy breeds exhibit some of the same features seen in the TCT dog, such as shortening and bowing of the limbs, a truncated rostrum, tooth crowding, and a notched foramen magnum.

Even without knowing the particular breed, the purpose and position of the TCT dog can still be discussed. As mentioned earlier, dogs inhabit a unique space within human society, and can exist in multiple positions, like that of a worker, companion, or even outcast. As mentioned in discussion of feists and terriers, small compact dogs were often used for hunting, varmint and rodent regulation, and guard duties. Based on its location near a coastal tavern, the TCT dog may have been intended as a ratter, had it survived. The rat-type terriers of the eighteenth century soon developed into individual breeds, such as the Manchester Terrier, the American Mountain Feist, and American Rat Terrier (AKC 2018; TKC 2009; UKC 2020). Alternatively, there are multiple attestations of small dogs being used as “spit jacks” or “turn spits”—dogs which were trained to walk on a wheel to turn a roasting spit in kitchens (Noel Hume 1978; Richardson 1847; Taplin 1803). These dogs were described in 1847 as “small, long-backed, cross-made dogs with the fore legs bent” (Richardson 1847:73), which closely resembles the physical condition of the TCT dog.

## Discussion

### Significance of Deposition

According to the Archaeological Data Recovery Synthesis Volume (Gallagher and Ritchie 1992), the 1994 site report (Gallagher et al. 1994), Craig Chartier’s (2016) architectural reassessment, the Massachusetts Historical Commission’s publication *Highway to the Past: The Archaeology of Boston’s Big Dig* (Lewis 2001), and Joseph M. Bagley’s book *A History of Boston in 50 Artifacts* (2016), the discussion and interpretation of the “cat” skeleton focuses heavily on ritual concealment, folklore, and witchcraft. As a cat, this skeleton functions as additional archaeological evidence to prove the presence of ritual concealment practices in colonial North America through the eitghteenth century. Chartier (2016) posits that it was placed to symbolically protect against rats and everyone involved in the original investigation seemed to agree that its burial, if intentional, may have been related to protection against witches (Gallagher et al. 1994; Gallagher and Ritchie 1992). However, when the identification is adjusted to a dog, it poses new questions of significance and meaning.

Any discussion about the cultural context of this dog’s burial must consider whether its status was as a pet, as a working dog, or as an animal with no value. In eighteenth-century England and its colonies, the attitudes toward dogs were undergoing a cultural shift towards seeing them not only as tools valued for their particular skills, but also as pets valued merely for their companionship (Blaisdell 1999; Gordon 2017; Thomas 2005). Enlightenment era thought had a certain degree of influence on the perceptions of animals as being equally sentient beings, with a move away from the church-influenced view of animal nature as being diametrically opposed to that of humans (Gordon 2017; Howard-Smith 2019; Thomas 2005). Rather than a simple dichotomous view of dogs as either strictly working animals or the frivolous pets of the wealthy, dogs filled a variety of social niches in urban environments like London, Boston, or even, Charlestown (Gordon 2017). With this cultural shift occurring, the TCT dog may very well have been a beloved pet, which accidentally died or had to be euthanized due to illness, leading to its intentional burial near the tavern. Alternatively, the value of a dog that may have worked as a ratter or turn spit at an ocean-side tavern cannot be overstated, and the loss of a potential working dog may also have afforded it an intentional burial. The final possibility is that this dog had no intrinsic value to the inhabitants of the tavern, and was either culled from an unwanted litter of puppies, or was even the victim of cruelty or sport.

The analysis of animal remains from the Great House/Three Cranes Tavern site was conducted by Dr. Richard Meadow and others at the Harvard University zooarchaeology laboratory; however, only bone material from the major privy and midden features on the site were analyzed (Meadow et al. 1994). The exclusion of Feature 193 is what most likely led to the persistent misidentification of the dog discussed in this report, as it likely would have been correctly identified if analysed at the time. A review of the faunal appendix published as part of the Gallagher et al. (1994) report showed 34 remains which were identified as *Felis catus,* all of which were found in privy features on the site. While no additional dog remains were found in any of the privies, it appears that deposition of non-food domestic animals in privies was commonplace, and the placement of the TCT dog within an intentionally dug pit is anomalous.

### Cultural Considerations of Dog Deposition

When considering the cultural significance of the dog from the TCT site it is important to also consider the different human groups that lived and worked at the site. From 1720-50s, when the dog was most likely interred in Feature 193, there were several distinct sociocultural groups present: the British colonial Long family and their descendants; the unnamed and briefly mentioned enslaved Africans that were known to be on the property at various points; the local Pawtucket Confederation of the Abenaki and other Indigenous North Americans, tavern patrons who may have been from any number of intersecting class and social groups; and the laborers, who Nathaniel Brown employed to undertake the extensive renovations to the property (Gallagher et al. 1994). Each group brought with them the specific beliefs and *habitus* which defined the way they navigated and experienced the world (Bourdieu 1977). With African, proto-American, European, and Indigenous religious and ritual systems all potentially at play at the TCT site, it is initially a daunting task to approach the potential “meaning” behind this dog burial, though reasonable assumptions can be made.

In 1639, a Pawtucket Squaw Sachem—sometimes called the “Squaw Sachem of Misticke”—whose name has been lost to history and is only defined by her relationship to her late husband Nanepashemet, deeded a large parcel of land to several prominent English colonists of the Massachusetts Bay Colony, including Governor Winthrop (Chapman 1936). This was a legally binding contract on both sides—although questions of coercion cannot be ignored—and resulted in the complete ownership of Mishawum – renamed Charlestown by colonists – by the Massachusetts Bay Colony. By the mid-eighteenth century, pressure by the English colonists had forced the disintegration of organized tribal presence within Charlestown. However, at least 15 Indigenous soldiers from the Mashpee Wampanoag, the Tunxis, the Hassanamisco Nipmuc, the Mohegan, and the Pequot tribes fought alongside Charlestown residents in the 1775 Siege of Boston and it stands to reason there were more Indigenous individuals who continued to live and work in the area during the eighteenth century (National Park Service 2015). This being said, dog interments made by culturally similar Indigenous groups in northeastern Massachusetts were generally restricted to either dogs buried alone with evidence for butchery, or dogs buried with human beings (Kerber 1997), neither of which match this individual circumstance.

Possible African presence and influence on the TCT property is a bit less clear, mostly due to historical erasure of enslaved Africans in Massachusetts. John Long and his wife Mary owned an enslaved girl with no recorded name, who lived and worked on the property at least until John’s death in 1683, but may have continued living with Mary on the property until her death in 1730 (Gallagher et al. 1994). The 1994 site report mentions other “servants” being at the property during the early eighteenth century, but does not identify their race until the mention of Zipporah, an enslaved black woman owned by Nathaniel Brown (Gallagher et al. 1994). Zipporah was present on the property from Brown’s purchase of it in 1746, but nothing further is reported about her except that she married a man named Cesar in 1757 (Gallagher et al. 1994). The presence of enslaved and free Africans at the Three Cranes Tavern property during the 1720-50s, when the dog was buried, broadens the scope of cultural groups which may have been responsible for placing it within its pit. There is immense variety to be found within the European and African spiritual worldviews that came together in Charlestown in the eighteenth century, but there is evidence for intentional burial of spiritually important items within both broad systems of belief.

### Ritual Concealment

Archaeological evidence for ritual concealment or caching of spiritually important items is present at sites associated with enslaved and free African communities and with European settlers (Augé 2013, 2014; Costello 2014; Fennell 2000, 2014; Galke 2000; Jones 2000; Leone et al. 2014; Leone and Fry 1999; Manning 2012, 2014; Reeves 2014; Wilkie 1997). In her 2014 paper, “The Material Culture of Ritual Concealment in the United States,” M. Chris Manning offers a helpful typology for understanding these past interpretations. She (Manning 2014:52) defines ritual concealment as a “deposit of one or more artifacts deliberately hidden within the structure of a building as part of a magico-religious or secular folk ritual.” Concealed items found at eighteenth-century archaeological sites in northeastern North America range from crystals, photographs, shoes, and ceramics, to mummified cats and animal bone assemblages. The explanations for such concealments include European foundation sacrifices going back centuries, English anti-witchcraft charms, and domestic protection rituals from both West Africa and the British Isles.

Foundation deposits were intended to offer general protection over a structure, and have been associated with those in the building trade of Europe by secondary historical sources like Jacob Grimm and G. W. Speth (Grimm 2014 [1835]; Speth 1894). Extensive archaeological evidence for foundation deposits in North America are reported by C. Riley Augé in her 2013 dissertation on protective magic used by Anglo-Europeans in seventeenth- to eighteenth-century New England. Augé (2013) provides a systematic review of the types of protective rituals seen within these contexts, and specifically discusses those involving faunal remains like cats and dogs. In her discussion of these deposited faunal remains, Augé (2013:274) highlights some of the typical locations where bones are concealed, including placing them “around doors, under floors, [and] buried in ground near a threshold.” These foundation deposits generally happen at the time of construction, or shortly before, and would be initiated by the homeowners or builders. Augé (2013) observes that some of these archaeological deposits of faunal material are directly associated with anti-witchcraft protection, especially cats, and this is clearly the lineage of interpretation that contributed to the TCT dog being related initially directly to witchcraft rituals (Augé 2013; Bagley 2016; Chartier 2016; Gallagher et al. 1994; Gallagher and Richie 1992Lewis 2001). If it was a foundation deposit, the TCT dog may have been intentionally placed to provide a degree of protection against anything from witchcraft and bad luck, to domestic pests and illness.

Related to these foundation deposits are the domestic protection rituals that are generally conducted by the inhabitants of a house– which could be owners, visitors, tenants, servants, or enslaved persons (Augé 2013, 2014; Manning 2012, 2014). The most famous examples of these in North America include concealed cats and concealed footwear, which are generally found within the walls or eaves of a house and are almost exclusively associated with Anglo-European contexts (Manning 2012, 2014). These domestic protection deposits can also be associated with what are sometimes called spiritual caches or middens, which are more commonly buried in floors or other ground-level contexts (Augé 2013). These caches often involve multiple types of items, with mixtures of broken ceramics, crystals, beads, bones, bent pins, and coins all attested in the archaeological record (Augé 2013; Manning 2012). There are some Anglo-European examples of these spiritual caches (Augé 2013; Manning 2012, 2014), but some of the most compelling examples are associated with enslaved or free Africans (Fennell 2000, 2014; Galke 2000; Jones 2000; Leone et al. 2014; Leone and Fry 1999; Reeves 2014; Wilkie 1997).

**Fig. 8:**
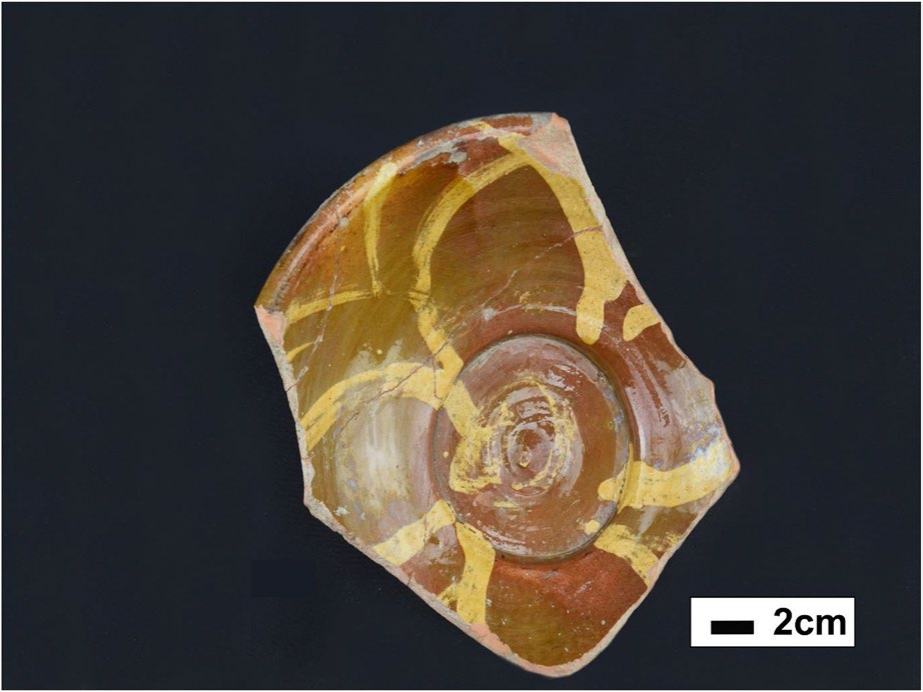
Top view of a slip-trailed redware bowl, dated between 1725–40 (by author)

**Fig. 9:**
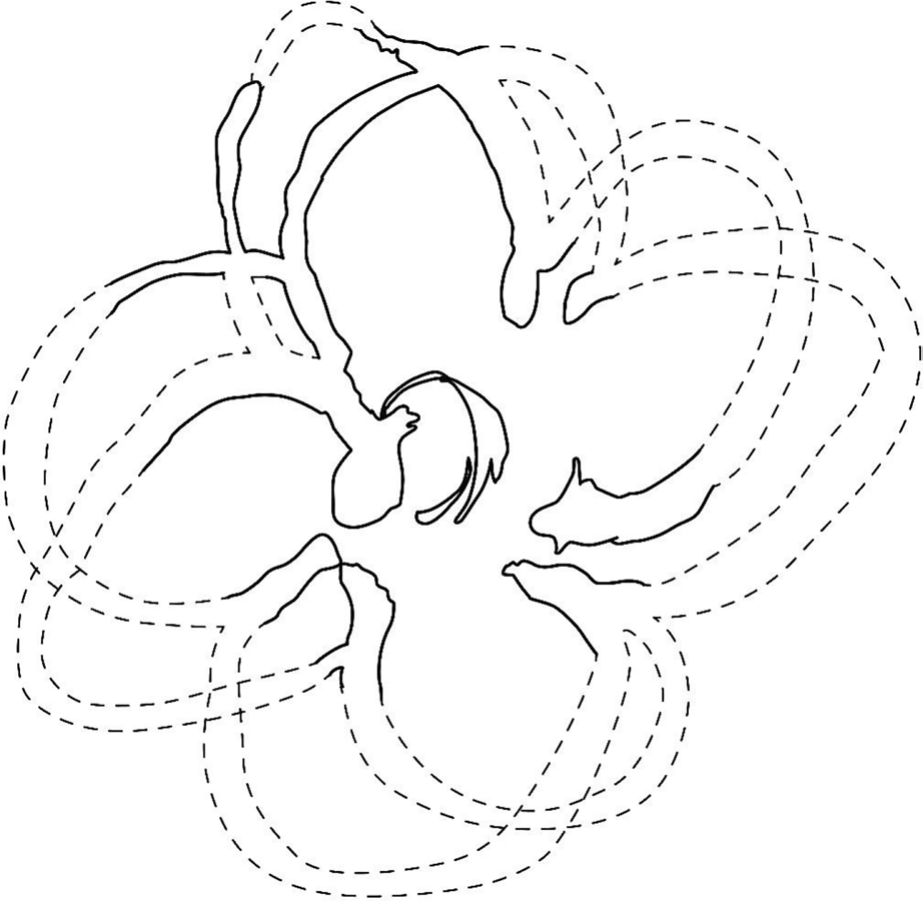
Artistic rendering of redware bowl slip-trailed design, extrapolated from present design (by author and Micaela Brody)

Often referred to as *minkisi* assemblages when discussing African Diaspora sites that have connections to Central African BaKongo cultures, these caches involve the creation of a *nkisi,* a “powerful religious object that, when activated by a spirit manifested in a three-dimensional object, can be used for healing or other medicinal purposes” (Martinez-Ruiz 2013:149). Part of BaKongo spiritualism, the *nkisi* may take the form of anthropomorphic or zoomorphic sculpture, but equally as commonplace are vessels like hollow gourds, baskets, shells, bottles, cauldrons, and pottery. These assemblages can involve multiple powerful *nkisi* objects, and in some areas of Central Africa are called *Kiniumba kia Mbumba,* which, according to Martínez-Ruiz (2013:171), “represents the act of gathering many things together for a specific purpose.” One specific *Kiniumba kia Mbumba,* called *kangadi a nzo*, involves the burial of objects in front of the main door of a building, sometimes in a redware bowl (Martinez-Ruiz 2013). There is evidence from other eighteenth-century sites like the Carroll House in Annapolis, Maryland that some ritual caches may represent a North American continuation of Central African BaKongo *minkisi* assemblages (Galke 2000; Jones 2000). The association of the TCT dog with a partial redware bowl may indicate it was intended as this kind of cache deposit, and the redware bowl itself may have more significance than initially believed. Figure 8 shows the mended redware bowl, and Fig. 9 shows the extant features and an artist’s concept of the complete four-petaled slip trailed design.

**Fig. 10:**
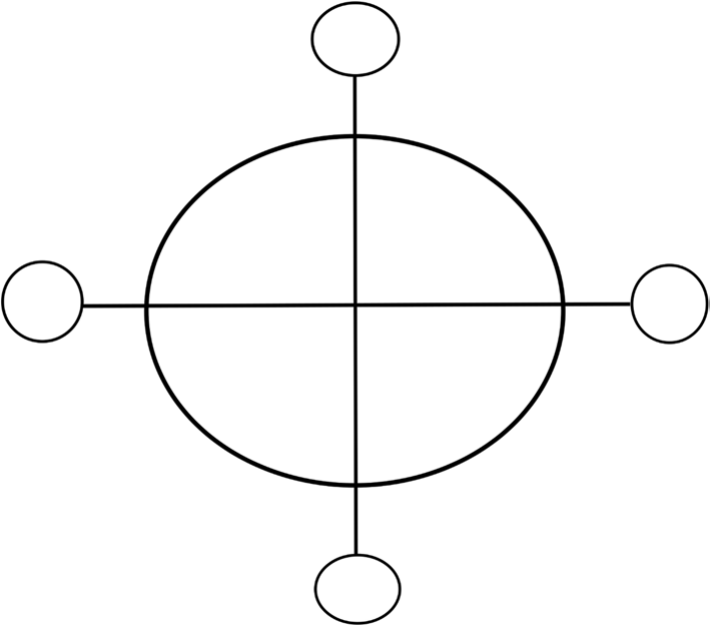
Simplified rendering of the *dikenga* or BaKongo cosmogram, after Fennell, 2003 (by author)

The slip-trailed design on this bowl is fairly unique, and was most likely produced at the nearby Parker Harris potteries, or one of many other potteries in Charlestown, many of which either had enslaved or free Africans working as potters (Hardesty pers. comm.; Pendery 1985; Watkins 1950). BaKongo religious symbolism is a complex and rich domain, with much emphasis placed on the dikenga cosmogram (Fig. 10) and its four structural elements, often represented with cross or starlike imagery (Fennell 2000, 2014; Gundaker 2011; Joseph 2011, 2017; Martinez-Ruiz 2013). The cosmogram itself is comprised of four cardinal points, which represent transitions between life stages as well as the living and spiritual world (Fennell 2000, 2014; Martinez-Ruiz 2013). Extensive records of graphical writing and pottery marks and designs used across Central African and African Diaspora communities in the Caribbean and North America have been compiled by researchers like Christopher Fennell and Bárbaro Martínez-Ruiz, showing the vast diversity of symbols which incorporate the dikenga elements. Researchers like Grey Gundaker (2011:176) stress the importance of seeing the dikenga cosmogram as having an unfixed form, describing, “change, mixture and innovation” as a “key premise of dikenga ideology.”

With this unfixed aspect of the dikenga cosmogram in mind, one possible interpretation of the four-petal slip trailed design on the redware bowl is as an embodiment of the four dikenga elements. Add to this the fact that, within BaKongo belief systems, the earth itself is viewed as a container for all the essential aspects of life and spiritual expression, then the connection between pottery and spiritually important vessels is even clearer (Martinez-Ruiz 2013). It is certainly possible that the unique design was produced by an enslaved or free African person practicing a form of BaKongo spirituality. This may then have been placed with the body of a recently deceased dog as a *kangadi a nzo* assemblage, perhaps even alongside plant materials or other organic remains which did not remain in the archaeological record. Ojoade (1990) reports that in at least one Nigerian myth, dogs ally themselves against all other animals alongside humans, and there are some concrete, albeit scattered, references within archaeological and anthropological literature assigning spiritual importance to dog bones within North American African Diaspora contexts (Greer 2016; Ojoade 1990; Russell 2011).

## Conclusions

**Fig. 11:**
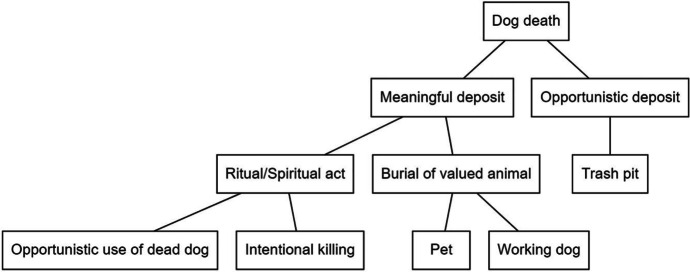
Flow chart showing potential deposition scenarios and motivations (by author)

The exact meaning of the dog’s placement in the intentionally dug pit is still unknown, and may never be revealed, but I have presented several explanations in this work. The most element of interpretation here involves whether this dog was intentionally killed for a ritual or otherwise meaningful purpose, or if its deposition was opportunistic. Figure 11 traces the possible interpretations of the dog’s placement in Feature 193, the intentionally dug pit below the newly laid eighteenth-century foundation at the Three Cranes Tavern.

If deposited for a ritual purpose, this dog may be an example of a foundation deposit made by builders, intended to protect the tavern additions from bad luck and structural faults through magical means. Alternatively, it may have been intended to protect the tavern from pests, illness or witchcraft, placed there by a member of the Long family or Nathaniel Brown himself. With several enslaved Africans being present at the TCT throughout the years there is also the possibility that the dog and redware bowl functioned together as a *kangadi a nzo* assemblage, providing protection for those inhabiting the domestic spaces within the tavern. While the trauma evident on the skull makes it likely that the dog was intentionally killed, the person who did so may not have been the same person who deposited it in the pit by the tavern’s southern entrance. This could represent either an opportunistic usage of a recently dead dog, or the dog may have been intentionally killed with a ritual deposit in mind. If not used for a ritual or spiritual purpose, the TCT dog may represent an intentional burial, of either a companion or future working animal which had to be euthanized due to illness or deformity. There is some question, however, as to why a pit for this animal would be dug underneath the area designated for the new tavern foundation, potentially undermining the structural integrity of the building. Additionally, if some kind of congenital disorder was present and apparent from birth, why allow the animal to suffer for nearly eight months if it was intended to be a pet or working dog?

Whether the skeleton from the TCT represents part of a building or protection ritual, a spiritual cache, a culled animal, or the intentional burial of a pet, it was accepted by all previous authors that there may be great significance associated with its demise, especially in the context of so much folkloric and archaeological evidence. However, when the identification of this specimen changes from a cat to a dog, some of this significance also changes. The impact of this reidentification on cultural interpretations is meaningful, as there is far more evidence for ritual burial of cats in North America than dogs (Augé 2013, 2014; Gallagher et al. 1994; Manning 2012, 2014). Additionally, this is the first osteobiography of its kind for an eighteenth-century dog skeleton and provides more direct evidence for the importance of understanding animal deposits, as called for by Tourigny et al. (2016) in their investigation of a nineteenth-century dog from Toronto.

Osteobiography lets us attempt to read a skeleton as a text; however, translation and interpretation are still dependant on the reader in many ways. Overton and Hamilakis (2013) critiqued some of these interpretation issues, noting that the established ontologies of social zooarchaeology rely heavily on positioning animals only as beings or objects to be exploited by humans. Most osteobiographies operate under the same principle, and it is difficult to separate the true “biography” of this dog as an individual, from the human-derived categories applied to it as an “associated bone group” or “special animal deposit.” Biomolecular analysis may shed more light on the TCT dog’s short life, with stable isotopes able to clarify aspects of diet and life history, a full palaeopathology assessment able to more clearly define disease and injury impacts, and DNA analysis useful for approximating specific dog breeds (Hosek and Robb 2019; Morris, 2011; Russell, 2011; Tourigny et al. 2016; Zouganelis et al. 2014). However, even after the application of such scientific analysis, the fact remains that meaning and interpretation has been, and continues to be, assigned to this dog by a bewildering multitude of people – the people responsible for the death of this dog; those who deposited it; anyone who knew of the deposition both during and after the act; the original excavators in the field; later writers who never laid eyes on the dog; myself, first as a fledgling zooarchaeologist volunteering in the City of Boston Archaeology lab and now as an established researcher; and, finally, you, the reader. When considering the impact of this dog it is not enough to just focus on how it experienced its short life, but how it continues to be a social actor even long after death.

Through careful consideration of the evidence outlined above, I conclude that the articulated skeleton found at the CSAD archaeological project’s excavation of the TCT was initially as a cat when it was first recovered in 1985–87. This misidentification was most likely based on the overall small size of the skeleton and roundedness of its incomplete juvenile skull, leading excavators and later researchers to identify it as a cat when it was, in fact, the remains of a small dog between 6.5 and 7.5 months of age at its time of death. I also concur with previous researchers that the deposition of this dog implies a degree of intentionality that reflects at least some cultural significance. Archaeological evidence may not conclusively prove whether the dog was intended to protect against witches, illness or pests, but it does imply that the person responsible for placing it in the pit with a redware bowl had some reason for doing so – whether mundane or magical.

## References

[CR1] AKC - American Kennel Club. (2018). *Terrier Group Breed Standards*. American Kennel Club, New York.

[CR2] Ameen C, Hulme-Beamn A, Evin A, Germonpre M, Britton K, Cucchi T, Larson G, Dobney K (2017). A landmark-based approach for assessing the reliability of mandibular tooth crowding as a marker of dog domestication. Journal of Archaeological Sciences..

[CR3] Andersone Z, Ozolins J (2000). Craniometrical characteristics and dental anomalies in wolves *Canis lupus* from Latvia. Acta Theriologica.

[CR4] Andreis, M. E., Polito, U., Veronesi, M. C., Faustini, M., Di Giancamillo, M., and Modina, S. C. (2018). Novel contributions in canine craniometry: anatomic and radiographic measurements in newborn puppies. *PloS One* **13**(5): e0196959. 10.1371/journal.pone.0196959 PMC5940217 29738556

[CR5] Augé, C. R. (2013). *Silent Sentinels: Archaeology, Magic, and the Gendered Control of Domestic Boundaries in New England, 1620–1725*. Doctoral dissertation, University of Montana. https://scholarworks.umt.edu/etd

[CR6] Augé CR (2014). Embedded implication of cultural worldviews in the use and pattern of magical material culture. Historical Archaeology.

[CR7] Bagley JM (2016). A History of Boston in 50 Artifacts.

[CR8] Bellei E, Ferro S, Zini E, Gracis M (2019). A clinical radiographic and histological study of unerupted teeth in dogs and cats: 73 cases (2001–2018). Frontiers in Veterinary Science.

[CR9] Benecke N (1987). Studies on early dog remains from northern Europe. Journal of Archaeological Science.

[CR10] Blaisdell JD (1999). The rise of man’s best friend: the popularity of dogs as companion animals in late eighteenth-century London as reflected by the dog tax of 1796. Anthrozoös.

[CR11] Blunt-Lytton JADW (1911). Toy Dogs and Their Ancestors, Including the History and Management of Toy Spaniels, Pekingnese, Japanese, and Pomeranians.

[CR12] Bockelmann, H. (1920). *Untersuchungen an Wolfsbastarden nach Ziichtungsversuchen im Haustiergarten zu Halle*. Doctoral dissertation, Martin-Luther-Universität-Halle-Wittenberg. Halle.

[CR13] Bourdieu P (1977). Outline of a Theory of Practice.

[CR14] Breen, E. E. (2013). *The Revolution before the Revolution? A Material Culture Approach to Consumerism at George Washington’s Mount Vernon, VA*. Doctoral dissertation, University of Tennessee, Knoxville.

[CR15] Caius, J. (2008 [1570]). *De Canibus Britannicis (Of Englishe Dogges).* Fleming, A.(trans.), Project Gutenberg.

[CR16] Calce SE, Rogers TR (2007). Taphonomic changes to blunt force trauma: a perimortem study. Journal of Forensic Science.

[CR17] Carmichael, L. E. (2005). An annotated historical account of canine parvovirus. *Journal of Veterinary Medicine. B, Infectious Diseases and Veterinary Public Health* **52**(7–8): 303–311. 10.1111/j.1439-0450.2005.00868.x 16316389

[CR18] Chapman HS (1936). History of Winchester Massachusetts.

[CR19] Chartier, C. S. (2016). *An Archaeological Reevaluation of the Great House/Three Cranes Tavern (1629–1775) Charlestown, Massachusetts*. Plymouth Archaeological Rediscovery Project, Plymouth, MA.

[CR20] Clutton-Brock J, Brothwell D, Higgs E (1963). The origins of the dog. Science in Archaeology: A Comprehensive Survey of Progress and Research.

[CR21] Costello J (2014). Tracing the footsteps of ritual: concealed Footwear in America. Historical Archaeology.

[CR22] Coulson A, Lewis N (2002). An Atlas of Interpretative Radiographic Anatomy of the Dog & Cat.

[CR23] Deetz JF (1999). In Small Things Forgotten: An Archaeology of Early American Life.

[CR24] Donald D, Stotik J (1992). Feist or fiction? The squirrel dog of the southern mountains. Journal of Popular Culture.

[CR25] Done, S., Goody, P., Evans, S., and Stickland, N. (2009). *Color Atlas of Veterinary Anatomy, Volume 3, The Dog and Cat.* Elsevier, Maryland Heights, MD.

[CR26] Drury, W. D. (1903). *British Dogs: Their Points, Selection and Show Preparation*. Upcott Gill, London.

[CR27] Evans HE, De Lahunta A (2012). Miller’s Anatomy of the Dog.

[CR28] Ewonus P (2011). Social zooarchaeology of a Northwest Coast house. Journal of Island and Coastal Archaeology.

[CR29] Fennell CC (2000). Conjuring boundaries: inferring past identities from religious artifacts. International Journal of Historical Archaeology.

[CR30] Fennell CC (2014). Artifacts to invoke, direct, and deflect. Historical Archaeology.

[CR31] Frothingham, R. (1845) *The History of Charlestown, Massachusetts*. Charles C. Little and James Brown, Boston, MA.

[CR32] Fulton A, Fiani N, Verstraete F (2014). Canine pediatric denstistry. Veterinary Clinics of North American Small Animal Practice.

[CR33] Galke LJ (2000). Did the Gods of Africa die? A re-examination of a Carroll House crystal assemblage. North American Archaeologist.

[CR34] Gallagher, J., Boros, L., DePaoli, N. K., Turner, A., and Fitzgerald, J. (1994). *Archaeological Data Recovery City Square Archaeological District Central Artery North Reconstruction Project Charlestown, Massachusetts. Volume VII (No. 50–57)*. The Public Archaeology Laboratory, Pawtucket, RI.

[CR35] Gallagher J, Ritchie D (1992). Central Artery North Reconstruction Project Charlestown, Massachusetts.

[CR36] Geiger M, Gendron K, Willimitzer F, Sanchez-Villagra MR (2016). Unaltered sequence of dental, skeletal, and sexual maturity in domestic dogs compared to the wolf. Zoological Letters.

[CR37] Geller P, Stodder ALW, Palkovich AM (2012). From cradle to grave and beyond: A Maya life and death. The Bioarchaeology of Individuals.

[CR38] Gerdin JA, McDonough SP (2013). Forensic pathology of companion animal abuse and neglect. Veterinary Pathology.

[CR39] Germonpré M, Sablin MV, Laznickova-Galetova M, Despres V, Stevens RE, Stiller M, Hofreiter M (2015). Palaeolithic dogs and Pleistocene wolves revisited: A reply to Morey (2014). Journal of Archaeological Science.

[CR40] Gordon R (2017). From pests to pets: social and cultural perceptions of animals in post-medieval urban centres in England (AD1500 – 1900). Papers from the Institute of Archaeology.

[CR41] Grant, A. (1984). Animal husbandry. In Cunliffe, B. (ed.), *Danebury: An Iron Age Hillfort in Hampshire. Volume 2. The Excavations 1969–1978: The Finds.* Council for British Archaeology Research, Report 52, London, pp. 496–548.

[CR42] Greer MC (2016). Contextualizing canines, a dog burial, and enslaved life on a Virginia Plantation. Journal of African Diaspora Archaeology and Heritage.

[CR43] Grimm, J. (2014 [1835]). *Deutsche Mythologie (Teutonic Mythology)*. Stallybrass, J. S. (trans.), Cambridge University Press, Cambridge.

[CR44] Grunberg, W. (2018) Phosphorus and Vitamin D in dogs. Merck Veterinary Manual- Merck, Sharp & Dohme Corp. https://www.msdvetmanual.com/dog-owners/bone,-joint,-and-muscle-disorders-of-dogs/disorders-associated-with-calcium,-phosphorus,-and-vitamin-d-in-dogs#:~:text=An%20excess%20of%20calcium%20has,meat%20diets%20commonly%20develop%20rickets. Accessed 6 August, 2021.

[CR45] Gundaker G (2011). The Kongo cosmogram in historical archaeology and the moral compass of Dave the potter. Historical Archaeology.

[CR46] Harasen G (2009). Feline orthopedics. *The*. Canadian Veterinary Journal..

[CR47] Harcourt RA (1974). The dog in prehistoric and early historic Britain. Journal of Archaeological Science.

[CR48] Hedges REM (2002). Bone diagenesis: an overview of processes. Archaeometry.

[CR49] Hill, J. D. (1995). *Ritual and Rubbish in the Iron Age of Wessex*. British Archaeological Report, British Series 242, Oxford.

[CR50] Hillson S (1990). Teeth.

[CR51] Hillson S (1992). Mammal Bones and Teeth: An Introductory Guide to Methods of Identification.

[CR52] Hosek L, Robb J (2019). Osteobiography: a platform for bioarchaeological research. Bioarchaeology International.

[CR53] Howard-Smith S (2019). Mad dogs, sad dogs and the ‘War against Curs’ in London in 1760. The British Journal for Eighteenth-Century Studies.

[CR54] Janssens L, Perri A, Crombe P, Van Dongeren S, Lawler D (2018). An evaluation of classical morphological and morphometric parameters reported to distinguish wolves and dogs. Journal of Archaeological Science: Reports.

[CR55] Jones L (2000). Crystals and Conjuring at the Charles Carroll House, Annapolis Maryland. African Diaspora Archaeology Newsletter.

[CR56] Joseph, J. W. (2011). “All of a Cross”: African potters, marks, and meanings in the folk pottery of the Edgefield District, South Carolina. *Historical Archaeology* **45**(2): 135–155.

[CR57] Joseph JW (2017). Crosses, crescents, slashes, stars: African-American potters and Edgefield District Pottery Marks. Journal of African Diaspora Archaeology and Heritage.

[CR58] Kerber JE (1997). Native American treatment of dogs in Northeastern North America: archaeological and ethnohistorical perspectives. Archaeology of Eastern North America.

[CR59] Kerr JW, Stimson AA (1909). The prevalence of rabies in the United States. Journal of the American Medical Association.

[CR60] Knowler SP, Galea GL, Rusbridge C (2018). Morphogenesis of canine Chiari malformation and secondary syringomyelia: disorders of cerebrospinal fluid circulation. Frontiers in Veterinary Science.

[CR61] Kranioti E (2015). Forensic investigation of cranial injuries due to blunt force trauma: current best practice. Research and Reports in Forensic Medical Science..

[CR62] Kupczynska M, Czubaj N, Barszcz K, Sokolowski W, Czopowicz M, Purzyc H, Dzierzecka M, Kinda W, Kielbowicz Z (2017). Prevalence of dorsal notch and variations in the foramen magnum shape in dogs of different breeds and morphotypes. Biologia.

[CR63] Leone MP, Fry G-M (1999). Conjuring in the Big House kitchen: an interpretation of African American belief systems based on the uses of archaeology and folklore sources. The Journal of American Folklore.

[CR64] Leone M, Knauf JE, Tang A, Ogundiran A, Saunders P (2014). Chapter 11: Ritual bundle in colonial Annapolis. Materialities of Ritual in the Black Atlantic.

[CR65] Lewis AEH (2001). Highway to the Past: The Archaeology of Boston’s Big Dig.

[CR66] Lewis G (2019). Musculoskeletal Development of the Puppy: Birth – Twelve Months. Animal Therapy Magazine..

[CR67] Manning, M. C. (2012). *Homemade Magic: Concealed Deposits in Architectural Contexts in the Eastern United States*. Master's thesis, Ball State University, Muncie, IN.

[CR68] Manning MC (2014). The material culture of ritual concealments in the United States. Historical Archaeology.

[CR69] Martinez-Ruiz B (2013). Kongo Graphic Writing and Other Narratives of the Sign.

[CR70] Meadow, R., Largy, T., and Stepien, J. (1994). Appendix Q: faunal analysis data mammal remains, Features 3, 5, 96, 98, 126, 142, 177, 188, 214, 235. In Gallagher, J., Boros, L., DePaoli, N. K., Turner, A., and Fitzgerald, J. (eds.), *Archaeological Data Recovery City Square Archaeological District Central Artery North Reconstruction Project Charlestown, Massachusetts. Volume VII (No. 50–7)*. Public Archaeology Laboratory, Pawtucket, RI.

[CR71] Modina SC, Andreis ME, Moioli M, Di Giancamillo M (2019). Age assessment in puppies: coming to terms with forensic requests. Forensic Science International.

[CR72] Moraitis K, Eliopoulos C, Spiliopoulou C (2009). Fracture Characteristics of Perimortem Trauma in Skeletal Material. The Internet Journal of Biological Anthropology.

[CR73] Morey DF (1994). The early evolution of the domestic dog. American Scientist.

[CR74] Morris, J. (2011). *Investigating Animal Burials: Ritual, Mundane and Beyond*. British Archaeological Reports Series 535, Oxford.

[CR75] National Park Service. (2015). Native Americans at Bunker Hill. National Park Service - Boston. https://www.nps.gov/bost/learn/historyculture/nativeamerican.htm; accessed September, 2020.

[CR76] Newton C, Nunamaker D (1985). Textbook of Small Animal Orthopaedics.

[CR77] Noël Hume, A. (1978). *Food*. Colonial Williamsburg Archaeological Series 9, Colonial Williamsburg Foundation, Williamsburg, VA.

[CR78] Ojoade JO (1990). Nigerian cultural attitudes to the dog.

[CR79] Olsen SJ, Olsen JW (1977). The Chinese Wolf, Ancestor of New World Dogs. Science.

[CR80] Onar V, Belli O (2005). Estimation of shoulder height from long bone measurements on dogs unearthed from the Van- Yoncatepe early iron age necropolis in Eastern Anatolia. Revue De Medicine Veterinaire.

[CR81] Onar V, Pazvant G, Gezer Ince N, Alpak H, Janeczek M, Kiziltan Z (2013). Morphometric analysis of the foramen magnum of Byzantine dogs excavated in Istanbul Yenikapi at the site of Theodosius Harbour. Mediterranean Archaeology and Archaeometry.

[CR82] Orton DC (2012). Taphonomy and interpretation: an analytical framework for social zooarchaeology. International Journal of Osteoarchaeology.

[CR83] Overton, N. J. and Hamilakis, Y. (2013). A manifesto for a social zooarchaeology: swans and other beings in the Mesolithic. *Archaeological Dialogues* **20**(2): 111–136.

[CR84] Pendery SR, Turnbaugh SP (1985). Ceramics and the colonial system: the Charlestown example. Domestic Pottery of the Northeastern United States, 1625–1850.

[CR85] Reeves, M. (2014). Mundane or spiritual?” In Ogundiran, A. and Saunders, P. (eds.), *Materialities of Ritual in the Black Atlantic*. Indiana University Press, Indianapolis, pp. 176-197.

[CR86] Ressel L, Hetzel U, Ricci E (2016). Blunt force trauma in veterinary forensic pathology. Veterinary Pathology.

[CR87] Richardson, H. D. (1847). *Dogs: Their Origins and Varieties, Directions as to their General Management and Simple Instructions as to their Treatment under Disease*. McGlashan, Dublin.

[CR88] Robb J, Hamilakis Y, Pluciennik M, Tarlow S (2002). Time and biography. Thinking through the Body: Archaeologies of Corporeality.

[CR89] Rusbridge C, McFadyen AK, Knower SP (2019). Behavioral and clinical signs of Chiari-like malformation-associated pain and syringomyelia in Cavalier King Charles spaniels. Journal of Veterinary Internal Medicine / American College of Veterinary Internal Medicine.

[CR90] Russell N (2011). Social Zooarchaeology: Humans and Animals in Prehistory.

[CR91] Saul FP (1972). The human skeletal remains of Altar de Sacrificios: an osteobiographic analysis. Papers of the Peabody Museum.

[CR92] Saul, F. P. and Saul, J. M. (1989). Osteobiography: a Maya example. In Iscan, M. Y. and Kennedy, A. R. (eds.), *Reconstruction of Life from the Skeleton*. Alan R. Liss, New York, pp. 287–301.

[CR93] Schoeneberger, P. J., Wysocki, D. A.,Benhem, E. C., and Soil Survey Staff. (2012). *Field Book for Describing and Sampling Soils, Version 3.0*. Natural Resources Conservation Service, National Soil Survey Center, Lincoln, NE.

[CR94] Shaw, V. (1881). *The Illustrated Book of the Dog*. Petter, Galpin, London.

[CR95] de Siqueira A, Cuevas SEC, Salvagni FA, Maiorka PC (2016). Forensic veterinary pathology: sharp injuries in animals. Veterinary Pathology.

[CR96] Smith, S. K. (2014). Firearms manufacturing, gun use, and the emergence of gun culture in early North America. *49th Parallel* **34**: 1–48.

[CR97] Speth, G. W. (1894). Builders’ rites and ceremonies: two lectures on the folk-lore of masonry. *Keble’s Gazette*: 55.

[CR98] Stefanowski T, Zablocki J (1969). Odmiany otworu nadklykcowego u kota domowego (Variations of the supracondyloid foramen in the domestic cat). Folia Morphologica.

[CR99] Stockard, C. R., Anderson, O. D., and James, W. T. (eds.) (1941). *The Genetic and Endocrinic Basis for Differences in Form and Behavior: As Elucidated by Studies of Contrasted Pure-Line Dog Breeds and their Hybrids, Volume 19*. Wistar Institute of Anatomy and Biology, Philadelphia.

[CR100] Studer, T. (1901). *Die praehistorischen Hunde in ihrer Beziehung zu den gegenwartig lebenden Rassen*. Zurcher und Furrer, Zurich.

[CR101] Sumner-Smith G (1966). Observations on epiphyseal fusion of the canine appendicular skeleton. Journal of Small Animal Practice.

[CR102] Sykes N (2014). Beastly Questions: Animal Answers to Archaeological Issues.

[CR103] Taplin W (1803). The Sportsman’s Cabinet, or, A Correct Delineation of the Various Dogs Used in the Sports of the Field: Including the Canine Race in General.

[CR104] Tarantola A (2017). Four thousand years of concepts relating to rabies in animals and humans, its prevention and its cure. Tropical Medicine and Infectious Disease.

[CR105] Thomas R, Pluskowski A (2005). Perceptions versus reality: changing attitudes towards pets in medieval and post-medieval England. *Just Skin and Bones? New Perspectives on Human-Animal Relations in the Historical Past* BAR International Series 1410.

[CR106] TKC - The Kennel Club. (2009). *Manchester Terrier Breed Standard*. The Kennel Club, London. https://www.thekennelclub.org.uk/services/public/breed/standard.aspx?id=3073; accessed August 2016.

[CR107] Tourigny E, Thomas R, Guiry E, Earp R, Allen A, Rothenburger JL, Lawler D, Nussbaumer M (2016). An osteobiography of a 19th-century dog from Toronto. Canada. International Journal of Osteoarchaeology.

[CR108] Uhl EW, Kelderhouse C, Buikstra J, Blick JP, Bolon B, Hogan RJ (2019). New World origin of canine distemper: interdisciplinary insights. International Journal of Paleopathology.

[CR109] UKC - United Kennel Club (2020). *Mountain Feist: Official UKC Breed Standard*. United Kennel Club. https://www.ukcdogs.com/mountain-feist; accessed August 2016.

[CR110] Unknown Author (1985–87a). *Central Artery North Phase III Field Notes*. City of Boston Archaeology Laboratory. West Roxbury, MA.

[CR111] Unknown Author (1985–87b). *Central Artery North Phase III Plan Records*. City of Boston Archaeology Laboratory. West Roxbury, MA.

[CR112] Velasco-Villa A, Mauldin MR, Shi M, Escobar LE, Gallardo-Romero NF, Damon I, Olson VA, Streicker DG, Emerson G (2017). The history of rabies in the western hemisphere. Antiviral Research.

[CR113] Von den Dreisch, A. (1976). *A Guide to the Measurement of Animal Bones from Archaeological Sites*. Peabody Museum of Archaeology and Ethnology, Cambridge, MA.

[CR114] Washington, G. (1770). George Washington Papers, Series 1, Exercise Books, Diaries and Surveys, 1745–99. Subseries 1B, Diaries 1748–1799: Diary, January 1. [Manuscript/Mixed material]. Retrieved from the Library of Congress, https://www.loc.gov/item/mgw1b.701/

[CR115] Watkins LW (1950). Early New England Potters and their Wares.

[CR116] Welker MH, Byers DA, McClure SB (2020). “I wanna be your dog”: evaluating efficacy of univariate and multivariate methods for differentiating domestic and wild canids in North America. International Journal of Osteoarchaeology.

[CR117] Wilkie LA (1997). Secret and sacred: contextualizing the artifacts of African-American magic and religion. Historical Archaeology.

[CR118] Yasar GQA (1998). Gunshot wounds to the skull: comparison of entries and exits. Forensic Science International.

[CR119] Young, A. (1846). *Chronicles of the First Planters of the Colony of Massachusetts Bay, 1623–1636*. Charles C. Little and James Brown, Boston, MA.

[CR120] Zouganelis GD, Ogden R, Nahar N, Runfola V, Bonab M, Ardalan A, Radford D, Barnett R, Larson G, Hildred A, Jones M, Scarlett G (2014). An old dog and new tricks: genetic analysis of a Tudor dog recovered from the Mary Rose wreck. Forensic Science International.

